# Perfectionism or Perfectionisms in Orthorexia Nervosa

**DOI:** 10.3390/nu15153289

**Published:** 2023-07-25

**Authors:** Caterina Novara, Sara Piasentin, Sofia Mattioli, Susanna Pardini, Eleonora Maggio

**Affiliations:** Department of General Psychology, University of Padova, Via Venezia 8, 35131 Padova, Italy

**Keywords:** orthorexia nervosa, perfectionism, eating disorder, obsessive–compulsive disorder, transdiagnostic, dieting

## Abstract

**Purpose:** Perfectionism is considered a transdiagnostic construct that characterises eating disorders (EDs) and obsessive–compulsive disorder (OCD) and that could also depict orthorexia nervosa (ON). The principal aim of this study was to investigate what dimensions of perfectionism mostly represent ON. Moreover, it was evaluated if dieting impacted the presence of orthorexic features and perfectionistic behaviour. **Methods:** The sample consisted of two groups—the “Diet” (n = 93), and the “No Diet” (n = 94) groups—composed of people with high and low orthorexic tendencies, respectively. Participants filled out self-report questionnaires to investigate orthorexic tendencies and different facets of perfectionism. **Results:** Analyses highlighted that people with high orthorexic tendencies showed higher perfectionistic features and that ON had a significant relationship with different facets of perfectionism. No interactions with diet were found. Therefore, no other differences were highlighted when the group type (Diet/No Diet) was considered. **Conclusions:** Regardless of diet, different facets of perfectionism characterise ON: perfectionism as a personality trait and perfectionism related to EDs and to obsessive–compulsive features. Our results evidenced that perfectionism could also be considered a useful construct in the conceptualization of orthorexia nervosa.

## 1. Introduction

Orthorexia nervosa (ON) is a psychopathological condition related to strict adherence to, and an excessive overconcern about, healthy diet. This disordered eating, described in 1997 by Steven Bratman, usually begins with a desire to improve well-being or to prevent/manage some clinical conditions [1]. The overconcerns about healthy food can lead to impairment in everyday functioning (e.g., social isolation, high levels of preoccupation with healthy eating, and a lot of time spent preparing food) and nutritional deficiencies [2,3].

To understand the core characteristics of ON, there have, over the years, been investigations into the relationships with other psychopathological constructs, emphasising strong similarities between ON and eating disorders (EDs) [4,5,6], and features analogous with obsessive–compulsive disorder (OCD) [7,8]. However, recent studies have highlighted more similarities between ON and EDs than ON and OCD [9,10,11]. The comparable features between ON, EDs, and OCD mostly concern perfectionism [6,12,13,14,15], which has been identified as a transdiagnostic feature in EDs [16] and in OCD [17,18]. Moreover, ON and EDs display disordered eating via strict control of food [16,19].

The literature highlights different types of perfectionism related to psychopathology as a general personality trait [20], a reflection of high and socially prescribed standards [21,22], and a belief that there is a perfect solution to every problem. This imposes the need to do things ideally, which can lead to severe consequences at the slightest mistake [23,24]. Frost et al. (1990) defined perfectionism as a multidimensional personality construct characterised by high personal standards of performance and tendencies for overly critical evaluations of one’s behaviour [20]. On the other hand, in the literature [25,26], two higher-order dimensions of perfectionism have been identified: perfectionistic strivings (PS), which refers to high personal standards and pursuit of perfection, and perfectionistic concerns (PCs), which include concerns about mistakes, fear of negative evaluations from others, and adverse reactions to imperfection. Only PCs shows positive relationships with maladaptive, dysfunctional, or unhealthy outcomes; moreover, PS is often related to both “adaptive” (e.g., active coping and positive effect) and “maladaptive” (e.g., passive coping and negative effect) outcomes according to personal goals and pursuits [26]. Two meta-analyses highlighted a relationship between perfectionism (total score, PC and PS) and EDs both in clinical sample of children and adolescents [27], and in a clinical sample of adults [28]. Moreover, the PC dimension shows an association with binge eating behaviours [29].

Considering the relationship between ON, EDs, and OCD characteristics, it is not surprising that perfectionist traits often occur in ON [11,13,14,15,30]. Yung and Tabri (2022) highlight that perfectionism is associated with ON features, and it is mostly in people who believe that their diet and lifestyle are healthy. Health-focused self-concept is empirically distinct from appearance-focused self-concept and ON characteristics show a positive association with both health-focused and appearance-focused self-concept. Consequently, the authors suggest that ON tendencies may reflect both health and appearance purposes [31].

Some studies have highlighted a relationship between maladaptive perfectionism (concern over mistakes) and ON [14,30]. It is also known that perfectionism is related to pursuing a diet in a dysfunctional way [32]. Moreover, in patients with obesity or on a diet, the only factor that specifically predicted higher levels of ON was perfectionism [15]. To the best of our knowledge, no studies have investigated the relationship between ON and the dimensions of PS and PC; while in EDs, PS is oriented almost entirely to physical appearance [33], in ON it could be related to a healthy life-style.

Therefore, the principal aim of our study was to investigate which perfectionism (PS or PC) also characterises ON. Furthermore, as a risk factor for developing ED and ON, we also intended to investigate whether dieting impacted the presence of orthorexic features and perfectionistic behaviour. Based on the present literature, our main hypotheses were:

(a) Individuals with high orthorexic characteristics could show more perfectionism than individuals with low orthorexic characteristics. In particular, orthorexic dieters could score higher on perfectionism scales than dieters without orthorexic features.
(b) Dieters with high orthorexic features could score higher on perfectionism scales than non-dieters with orthorexic features.

## 2. Methods

### 2.1. Participants

The total study group consisted of 187 adults, divided into two groups named “Diet” (n = 93), and “No Diet” (n = 94). The sociodemographic characteristics of the groups and comparisons between them are reported in Table 1.

The Diet group participants were consecutively admitted and voluntarily following a “zone diet”, a diet focused on low carbohydrate consumption, prescribed by a dietician in northern Italy. They filled out the questionnaires (received by their doctor) at home and then delivered them to the dietician. No participant was diagnosed with a psychiatric disorder or was taking psychotropic drugs. In this group, 53.8% were female (n = 50), mean age was 45.56 (SD = 12.85), and participants had a mean of 14.34 (SD = 3.35) years of school attendance. In addition, 25.8% were single or had a fiancé (n = 24), 7.50% were students (n = 7), and 51.61% had full-time employment (n = 48). The mean BMI was 24.65 (SD = 5.42).

The No Diet group was composed of 94 medical and nutrition-science students enrolled at the University of Padova and Verona (Italy) using snowball sampling. No participants were diagnosed with a psychiatric disorder, tok psychotropic drugs, or followed a diet at the time of administration. This group was composed of 65 females (69.10%), had a mean age of 22.60 (SD = 3.27), and had a mean of 16.09 (SD = 1.55) years of school attendance. In this group, 94.70% were single or had a fiancé (n = 89), and 2.10% had full-time employment (n = 2). The mean BMI was 21.84 (SD = 3.38).

The total sample, regardless of the diet, was divided into two groups named, respectively, “High EHQ” (n = 87) (including those who scored at or above the 90th percentile based on the total sample in the Eating Habits Questionnaire) and “Low EHQ” (n = 89) (EHQ percentile < 90).

The subjects participated voluntarily and were informed about the aims of the research. The anonymity and confidentiality of the data collected and the analysis in aggregate form were guaranteed. Before taking part in the study, the participants gave their written and informed consent. Subsequently, they responded to a self-reported questionnaire in a session that lasted about 30 min. All the measures were administered in counterbalanced order to avoid any effects of the order.

This study was part of a larger project and some research works had been published previously [9,11,15].

### 2.2. Measures

All participants filled out a demographic schedule to collect data about gender, marital status, age, years of school attendance, employment, BMI (kg/m^2^) (calculated on self-reported information about weight and height), and whether they were following a diet at the time of administration. Moreover, psychopathological characteristics were assessed with the following self-report questionnaires:

− Eating Habits Questionnaire (EHQ-21) [34], Italian version by [35]: a 21-item questionnaire to evaluate orthorexia nervosa on a four-point Likert scale divided into three scales: “Knowledge”, “Problems”, and “Feelings”. Psychometric properties (internal consistency and test–retest reliability) were good in the original and the Italian validation of the instrument. For the current study, Cronbach’s α displayed an excellent internal consistency for the total score (Cronbach’s α = 0.91) and the three scales.
− Multidimensional Perfectionism Scale (MPS) [20,36], Italian version by [37]: a 35-item questionnaire to assess perfectionism on a five-point Likert scale. The authors identified the dimension of positive strivings of perfectionism, with higher levels of planning and lower procrastination (expressed by the “Personal Standards” scale), and negative perfectionism, characterised by maladaptive concerns about actions (“Concern over Mistakes” and “Doubting of Actions” scales). The original and the Italian psychometric properties were good and, in the current study, internal consistency was good both for MPS Striving (Cronbach’s α = 0.86) and MPS Concerns (Cronbach’s α = 0.93).
− Eating Disorder Inventory-3 (EDI-3) [21], Italian version by [22]: a 91-item questionnaire used to assess symptoms and features of eating disorders and scored on a six-point Likert scale. The questionnaire is composed of twelve scales measuring eating disorder symptoms and general psychological features related to the development of EDs (which included “Perfectionism”). In both the original and Italian validation internal consistency and test-retest reliability were good. For the current study, “EDI-Perfectionism” showed good internal consistency (Cronbach’s α = 0.70), as did other subscales.
− Obsessive Beliefs Questionnaire (OBQ-46) [23,24], Italian version by [38,39]: a 46-item questionnaire that uses a seven-point Likert scale used to evaluate cognitive domains related to the development and maintenance of obsessive–compulsive disorder. It is composed of five subscales in which is included “Perfectionism”, a measure of the need to do things perfectly, and the perceived sense of failure when certain personal standards are not met. Psychometric properties (internal consistency, test-retest reliability, and convergent/discriminant validity) were good both in the original and Italian validation. This study showed excellent internal consistency for the “OBQ-Perfectionism” scale (Cronbach’s α = 0.92) and other dimensions.

### 2.3. Statistical Analysis

Quantitative statistical analyses were performed using the IBM Statistical Package for Social Sciences (SPSS) Version 29.0 software [40]. Cronbach’s alpha was assessed for all scales and subscales of each self-report questionnaire. Frequencies, means, and standard deviations were measured to explore sociodemographic features. The comparisons of discrete variables between groups were deployed based on the Chi-squared index.

To examine differences between and within subjects, the two groups were evaluated by a multivariate MANCOVA.

## 3. Results

### 3.1. Sociodemographic Characteristics

Groups differed in gender (χ^2^ = 4.67; *p* < 0.01), age (F_(2)_ = 297.82; η^2^ = 0.30; *p* < 0.001), and years of school attendance (F_(2)_ = 19.86; η^2^ = 0.07; *p* < 0.001). There were also differences in marital status (χ^2^_(2)_ = 92.95; *p* < 0.001), employment (χ^2^ = 149.89; *p* < 0.001), and BMI (F_(2)_ = 18.49; η^2^ = 0.10; *p* < 0.001). The Diet group was composed of more females, who were older, and had fewer years of school attendance than the No Diet group. Moreover, participants of the Diet group had more full-time employment and a higher BMI than the No Diet group. The Diet group was composed of more cohabiting partners than the No Diet group. MANCOVA was performed using all those variables as covariates, to control the sociodemographic differences between groups.

### 3.2. Perfectionism and Orthorexia Nervosa

Conducting ANOVA on group type (Diet/No Diet) and orthorexia nervosa (High and Low EHQ) we highlighted a statistically significant difference between the Diet (M = 51.16; SD = 8.92) and No Diet (M = 43.22; SD = 12.55) groups, with higher scores on the EHQ total for the Diet group (F_(1185)_ = 24.65; η^2^ = 0.118; *p* < 0.001).

Conducting MANCOVA on group type (Diet/No Diet) and orthorexia nervosa (High and Low EHQ) and controlling for some sociodemographic variables (gender, marital status, employment, age, years of school attendance, and BMI) we highlighted a single effect of the factor High/Low EHQ (F = 8.54; η^2^ = 0.17; *p* < 0.001) and a significant interaction between “group type” and High/Low EHQ (F = 6.51; η^2^ = 0.14; *p* < 0.001). In EDI-Perfectionism the No Diet group with High EHQ (M = 7.98; SD = 3.99) showed a statistically significant difference to the Diet Group with High EHQ (M = 4.87; SD = 2.47) and Low EHQ (M = 3.60; SD = 2.73) and with the No Diet group with Low EHQ (M = 3.83; SD = 2.71), with higher scores on the EDI-Perfectionism for the No Diet Group with High EHQ (F = 13.03; η^2^ = 0.07; *p* < 0.001).

### 3.3. Differences between High and Low EHQ in Perfectionist Traits

**Figure 1:**
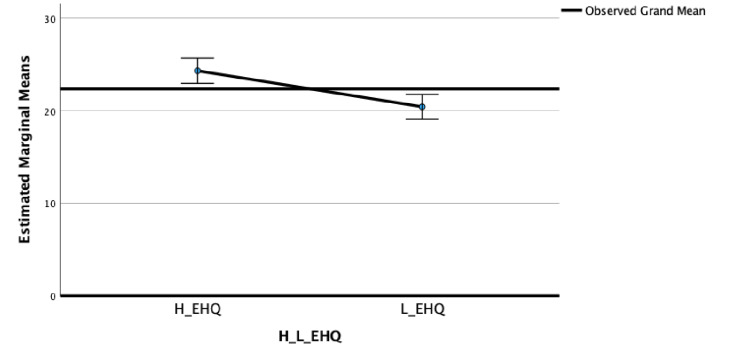
Means of Perfectionism Striving in High and Low EHQ groups.

**Figure 2:**
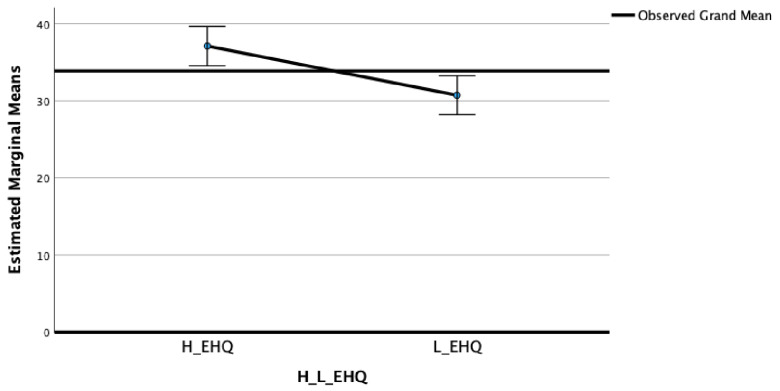
Means of Perfectionism Concerns in High and Low EHQ groups.

In the MPS dimensions “Striving” (F_(2)_ = 11.56; η^2^ = 0.07; *p* < 0.001) (Figure 1) and “Concerns” (Figure 2) (F_(2)_ = 9.20; η^2^ = 0.05; *p* < 0.01) (Figure 2), significant differences between High and Low EHQ were highlighted.

**Figure 3:**
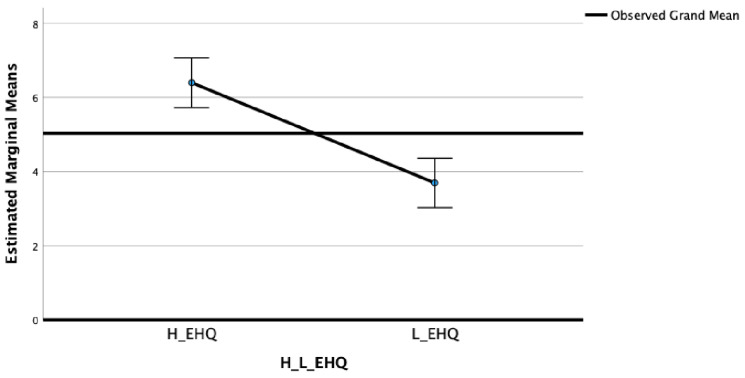
Means of EDI Perfectionism in High and Low EHQ groups.

**Figure 4:**
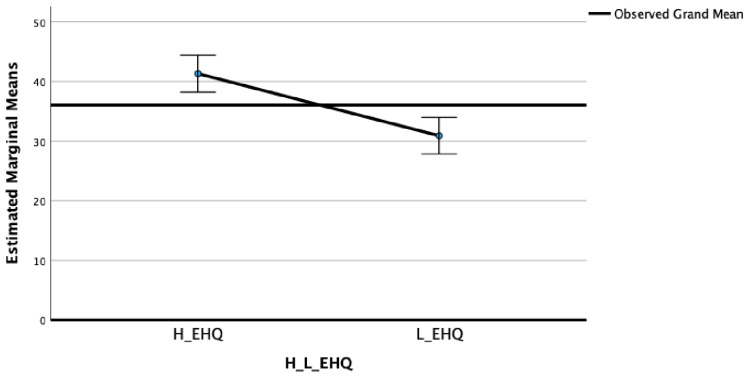
Means of OBQ Perfectionism in High and Low EHQ groups.

In the EDI Perfectionism subscale (F_(2)_ = 32.33; η^2^ = 0.16; *p* < 0.001) (Figure 3) and in the OBQ Perfectionism subscale (F_(2)_ = 16.13; η^2^ = 0.09; *p* < 0.001) (Figure 4), significant differences between groups were highlighted.

The post-hoc Bonferroni tests highlighted that the High EHQ group showed in MPS Striving (M = 24.23; SD = 6.00), MPS Concerns (M = 37.01; SD = 10.86), EDI Perfectionism (M = 6.37; SD = 3.63), and OBQ Perfectionism (M = 41.77; SD = 13.99) scales statistically significantly higher scores than the Low EHQ group (M = 20.49, SD = 6.62; M = 30.81, SD = 13.12; M = 3.72, SD = 2.71; and M = 30.45, SD = 14.72, respectively).

## 4. Discussion

Considering the relationship between ON, EDs, and OCD, the present study aimed to investigate better whether perfectionism, as a transdiagnostic construct, can also characterise other more recently studied and less defined disorders such as ON. In particular, we aimed to investigate what type of perfectionism (PC or PS) could characterise ON. Another aim was to investigate whether dieting ffected the presence of orthorexic characteristics and perfectionistic behaviour, being risk factors for the development of ED and ON.

In line with our hypothesis, people with high orthorexic tendencies showed higher scores on all the scales that investigated perfectionism (MPS-PC, MPS-PS, EDI-Perfectionism, and OBQ-Perfectionism).

To the best of our knowledge, this is the first study in which three facets of perfectionism, developed in different contexts, have been evaluated in ON. Specifically, perfectionistic concerns and perfectionism linked both with EDs and OCD are related to more negative outcomes [26] or are developed for the assessment of perfectionism in EDs [21] and OCD-related characteristics [23,24], while perfectionistic strivings display adaptive or maladaptive outcomes according to the goals people decide to pursue [26]. Perfectionistic strivings are related to the self-oriented pursuit of perfection and high personal standards in performance, while perfectionistic concerns regard concerns about making mistakes, fear of negative social evaluation due to not being perfect, doubts about actions, comparisons between one’s high standards and current performance, and presence of adverse reactions when something is not perfect [25]. Perfectionism linked with EDS is characterised by two aspects: “self-oriented perfectionism”, which refers to rigid personal standards of performance, and “socially prescribed perfectionism”, which is related to excessive requests from parents or teachers [21]. Lastly, perfectionism related to OCD is related to the idea that it is possible and mandatory to perform things in a perfect way to prevent the risk of even the smallest mistake, which could lead to severe consequences [23,24]. In people with ON, perfectionistic goals could be related to a long time spent choosing and preparing food [12] to prevent any eating-related mistakes; following strict dietary rules [12] due to high personal standards in performance; or a ritualised eating behaviour [12] consistent with the belief that is possible to follow perfect eating habits. Moreover, the fact that people with high orthorexic characteristics showed higher perfectionistic features is consistent with the conceptualization of perfectionism as a transdiagnostic construct for different disorders [16,17,18]. Taken together, these results could suggest that all these heterogeneous types of perfectionism characterise ON and the pursuit of healthy eating habits in a dysfunctional way—all perfectionisms characterise ON.

However, the hypothesis that orthorexic dieters show higher levels of perfectionism than those without orthorexic features has yet to be confirmed, and further study is needed. Specifically, at a qualitative level, higher levels of perfectionism emerge in orthorexic dieters than in dieters without orthorexic features but not in a statistically significant way, probably due to a low sample size.

Since in the present study, the Diet group showed significantly higher scores than the No Diet group on the EHQ questionnaire, we also investigated, in groups with orthorexic features, if perfectionism mainly characterised dieters rather than non-dieters. In spite of our hypothesis, no other differences were highlighted when the group type (Diet/No Diet) was considered; people with ON show more perfectionism regardless of being on a diet or not. In our study, there was no interaction with diet, probably because it is possible that it does not always characterise ON and because there are other factors deserving attention. Results highlighted that orthorexic dieters did not differ in Perfectionism Striving, Perfectionism Concerns, and Perfectionism related to OCD from orthorexic no-dieters, but differed in Perfectionism linked with EDs, which was higher in orthorexic non-dieters. These results could be counterintuitive; Fairburn considers perfectionism as a personality trait that has a key role in the development and maintenance of EDs, because it precedes the onset and persists after the recovery from the disease [16]. Our results highlight the presence of a group of people with high orthorexic tendencies and not on a diet that was composed of medical and nutrition science students. According to the literature, those students are most at risk of ON [41,42,43] and could deserve clinical attention for their higher levels of perfectionism (EDI-Perfectionism) which has a central role in developing EDs or ON. Regardless of diet, this group of students showed high orthorexic tendencies and high perfectionism.

To conclude, in line with our primary hypothesis, the results of our study suggest that people with high orthorexic tendencies show higher levels of different facets of perfectionism, related both to a personality trait, but also linked to obsessive–compulsive features and characteristics of EDs.

A limitation of the present study is that it was cross-sectional and there is a need to provide more information on the relationships of these factors over time. Longitudinal studies could be helpful in monitoring whether dieters with high orthorexic tendencies and high levels of perfectionism could present more psycho-physical sequelae than non-dieters with high orthorexic tendencies and high levels of perfectionism. Future studies should investigate what role perfectionism may have in following a diet, in relation to the goals that the person sets, such as seeking a high state of health or a healthy perception of the body. Another study limitation was the sample size of subgroups, thus statistically significant differences between these groups may have yet to emerge.

## 5. Conclusions

Scientific literature considers perfectionism as a transdiagnostic construct in EDs and OCD, and a common feature between EDs, OCD, and ON. In fact, the presence of perfectionist traits in EDs and OCD is well known, but they often also occur in ON.

To our knowledge, this is the first study where different types of perfectionism (as a personality trait, linked with EDs and OCD) have been evaluated in ON. Our study highlights that all these facets of perfectionism characterise ON, and it is independent of following a diet or not. Also, perfectionism can be considered a transdiagnostic construct in orthorexia nervosa. Moreover, our results evidence that medical and nutrition science students are deserving of clinical attention, as the presence in these people, regardless of following a diet or not, of higher levels of Ed-related perfectionism, has a central role in the development of EDs or ON.

## Figures and Tables

**Table 1:** Sociodemographic characteristics and comparisons between groups.

	Group Type	N (%) or M (SD)	Pearson Chi-Square or F	*p*	Partial η^2^
BMI	Diet	24.65 (5.42)	18.49	<0.001	0.10
	No Diet	21.84 (3.38)			
Gender (% female)	Diet	50 (53.8%)	4.67	<0.01	0.16
	No Diet	65 (69.1%)			
Marital status (% single or fiancé)	Diet	24 (25.8%)	92.95	<0.001	0.71
	No Diet	89 (94.7%)			
Employment (% full time)	Diet	48 (51.6%)	149.89	<0.001	0.90
	No Diet	2 (2.1%)			
Employment (% student)	Diet	7 (7.5%)			
	No Diet	91 (96.8%)			
Age	Diet	45.56 (12.85)	297.82	<0.001	0.30
	No Diet	22.60 (3.27)			
Years of school attendance	Diet	14.34 (3.35)	19.86	<0.001	0.07
	No Diet	16.09 (1.55)			

Note. M = mean; SD = standard deviation.

## Data Availability

The dataset used during the current study are available from the corresponding author on reasonable request.
